# PhysioKey: Edge-AI-Driven Physiological Key Agreement for Secure Body Area Networks

**DOI:** 10.3390/s26092605

**Published:** 2026-04-23

**Authors:** Mohammed Alnemari, Osamah M. Al-Omair

**Affiliations:** 1 Department of Computer Engineering, Faculty of Computing and Information Technology, University of Tabuk, Tabuk 71491, Saudi Arabia; 2 AIST Research Center, University of Tabuk, Tabuk 71491, Saudi Arabia; 3 Department of Management Information Systems, College of Business Administration, King Faisal University, Al-Ahsa 31982, Saudi Arabia; oalomair@kfu.edu.sa

**Keywords:** body area network, physiological key agreement, TinyML, edge AI, ECG, PPG, fuzzy commitment, healthcare IoT security

## Abstract

Body area networks (BANs) require secure intra-body communication, yet sensor nodes are too resource-constrained for conventional public-key cryptography, and pre-shared key schemes conflict with plug-and-play clinical workflows. This paper introduces PhysioKey, a TinyML-based key agreement framework that derives symmetric session keys from physiological signals without pre-shared secrets or trusted third parties. A lightweight 1D-CNN (6320 parameters, INT8-quantized, 31.2 KB flash) extracts embeddings from ECG and PPG windows on ARM Cortex-M4 class devices, which are reconciled through fuzzy commitment with BCH error-correcting codes. Patient-level 5-fold cross-validation on PTB-XL (500 patients, dual-ECG) achieves EER of 7.8%±0.8% with ROC AUC 0.978±0.004; on BIDMC (53 patients, ECG + PPG), a dual-encoder architecture reduces cross-modal EER to 30.6%±1.2%. Since standalone PhysioKey yields only 7–24 effective key bits, the recommended deployment mode is a hybrid PhysioKey + ECDH protocol providing 128-bit security while PhysioKey adds physical on-body authentication; standalone operation suits energy-constrained scenarios with its 27× advantage over ECDH. HKDF-SHA-256 post-processing yields session keys passing all six NIST SP 800-22 tests (≥96% at the 1024-bit level).

## 1. Introduction

Body area networks (BANs) enable continuous physiological monitoring through miniaturized sensors placed on or within the human body [1,2], with applications including ICU monitoring, post-operative recovery, and chronic disease management [3]. In a typical deployment, heterogeneous sensor nodes such as electrocardiogram (ECG) electrodes, photoplethysmogram (PPG) sensors, accelerometers, and temperature probes communicate wirelessly with a body-worn coordinator, which relays data to clinical systems [4].

Securing intra-BAN communication is both critical and difficult. Physiological data falls under strict regulations such as HIPAA and GDPR [5]. Interception or manipulation of BAN traffic could compromise patient privacy, falsify clinical readings, or disrupt therapeutic devices like insulin pumps [6]. Yet BAN sensor nodes operate under severe constraints: ARM Cortex-M class processors at 64–168 MHz, 256–512 KB flash, 64–128 KB SRAM, powered by coin-cell batteries of 100–300 mAh [7]. Traditional Public Key Infrastructure is impractical given the computational burden of asymmetric operations and certificate overhead [8].

Existing BAN key establishment approaches fall into three categories, each with notable shortcomings. *Pre-shared key* schemes require manual configuration or a trusted authority, creating deployment friction incompatible with dynamic clinical environments [9]. *Lightweight public-key* approaches based on Elliptic Curve Cryptography reduce cost relative to RSA but still demand energy-intensive point multiplications on constrained hardware [10]. *Physiological signal correlation* methods exploit the shared biological origin of signals measured at different body sites to derive common secrets [11,12,13]. These are appealing conceptually, but existing implementations rely on hand-crafted features (inter-pulse intervals, peak amplitudes) that exhibit limited discriminative power, high sensitivity to sensor placement, and no adaptation to individual patients [14].

No existing scheme provides an adaptive, learning-based key agreement mechanism that fits within BAN resource constraints while eliminating pre-shared secrets. TinyML, which refers to machine learning inference on microcontrollers with milliwatt power budgets, offers a path forward [15,16]. By deploying learned feature extractors on sensor nodes, patient-specific physiological patterns can be captured that are simultaneously correlated between co-located sensors and difficult for external adversaries to predict.

This paper introduces PhysioKey, an Edge-AI-driven physiological key agreement framework. The core idea is that a lightweight 1D-CNN, quantized to INT8 and occupying 31.2 KB flash, can extract embedding vectors from physiological signal windows that reflect the body’s shared physiological state while remaining robust to sensor noise and placement variation. These embeddings are reconciled through a fuzzy commitment scheme to produce symmetric session keys. The principal contributions are:

1. A TinyML-based physiological key agreement framework that uses a learned 1D-CNN feature extractor to derive correlated embeddings from ECG and PPG signals on Cortex-M4 class devices, enabling plug-and-play enrollment without pre-shared secrets.
2. A formal threat model for intra-BAN communication covering passive eavesdropping, active replay, impersonation, and model inversion attacks.
3. An honest characterization of the trade-off between key agreement success rate and effective key length, with entropy analysis that distinguishes the entropy of the quantized representation from the entropy of the source signal.
4. A dual-dataset evaluation with patient-level 5-fold cross-validation on PTB-XL (500 patients, dual-ECG, EER =7.8%±0.8%) and BIDMC (53 patients, synchronized ECG + PPG), demonstrating that a shared-encoder architecture fails on true cross-modal pairing (EER ∼48.3%), but a dual-encoder with modality-specific convolutional stacks reduces EER to 30.6%±1.2%.
5. Ablation studies quantifying the contribution of each loss component (contrastive, alignment, decorrelation), embedding dimension, and window length to key agreement performance.
6. A hybrid PhysioKey + ECDH protocol, recommended as the primary deployment mode, that combines on-body authentication with cryptographic key exchange, achieving 128-bit session keys while maintaining PhysioKey’s plug-and-play property. Standalone PhysioKey (7–24 effective key bits) is positioned as an energy-efficient alternative for scenarios where security requirements are modest.

The paper is organized as follows. Section 2 surveys related work. Section 3 formalizes system and threat models. Section 4 details the PhysioKey framework. Section 5 presents the security analysis. Section 6 reports evaluation results. Section 7 discusses limitations, and Section 8 concludes.

## 2. Related Work

This section surveys five related areas and identifies the gap motivating PhysioKey.

### 2.1. Key Distribution in Wireless Sensor Networks

Early key management schemes for wireless sensor networks established approaches that influenced BAN security. Eschenauer and Gligor [9] proposed probabilistic key pre-distribution where each node is preloaded with a random key subset from a global pool. While this eliminates online key exchange, it provides only probabilistic connectivity and requires a trusted authority. Chan et al. [10] extended this with *q*-composite pre-distribution, trading network scalability for improved capture resilience. Liu et al. [17] further developed polynomial-based pairwise key establishment, reducing storage overhead but retaining the assumption of a trusted key distribution center.

Zhu et al. [18] introduced LEAP+, which establishes four key types (individual, pairwise, cluster, and group) through pre-deployment material and localized post-deployment establishment. LEAP+ supports efficient revocation but depends on a static key hierarchy unsuited to BANs, where sensors are frequently repositioned during clinical workflows.

The fundamental limitation of all pre-distribution approaches is their assumption of deployment-time trust. In clinical BAN scenarios, a nurse may attach several sensors within minutes, and these must immediately establish secure channels without manual key entry or centralized coordination.

Modern BLE 5.0 LE Secure Connections [19] offer authenticated pairing without pre-shared keys via Numeric Comparison or Passkey Entry, but these methods require human interaction (visually confirming a 6-digit code or entering a passkey), which is impractical in high-turnover clinical environments where a single nurse may attach dozens of sensors per shift, and infeasible for unconscious or sedated patients who cannot participate in pairing confirmation. Out-of-band (OOB) pairing could leverage a side channel, but the BLE specification does not define a physiological OOB mechanism. PhysioKey can be viewed as providing a physiological OOB channel that enables fully automated, hands-free authenticated pairing without human intervention.

Vehicular and IoT networks have explored blockchain-assisted authentication as an alternative to symmetric pre-distribution. Alazzawi et al. [20] achieved efficient conditional anonymity with message integrity for VANETs, and Sutrala et al. [21] designed a batch verification scheme with conditional privacy for Internet of Vehicles deployments. These protocols rely on consensus mechanisms and infrastructure-level computation that exceed what BAN sensor nodes can support (Cortex-M4, coin-cell battery, kilobyte-scale memory). PhysioKey targets a fundamentally different operating point: autonomous, infrastructure-free key agreement on milliwatt-class devices.

### 2.2. Physiological Signal-Based Security

Exploiting the human body as a source of shared randomness for BAN key agreement was pioneered by Poon et al. [11], who showed that inter-pulse intervals (IPIs) measured simultaneously at different body locations exhibit sufficient correlation to serve as a common secret. Their scheme quantizes IPIs and uses error-correcting codes for reconciliation. While foundational, the reliance on a single hand-crafted feature makes it vulnerable to external estimation and degrades in patients with arrhythmias.

Venkatasubramanian et al. [12] proposed PSKA, extending IPI-based approaches with multiple features including peak amplitude and frequency-domain characteristics. PSKA reduces the number of signal windows needed for agreement but still relies on predetermined feature functions that do not adapt to individual physiology.

Rostami et al. [13] presented heart-to-heart (H2H), using ECG features for key agreement between an implantable device and an external programmer. H2H provides formal guarantees under the random oracle model but targets the implant-programmer scenario specifically, not the multi-sensor BAN setting.

Xu et al. [14] proposed Walkie-Talkie, using gait patterns from accelerometers for key agreement. This is inapplicable to bedridden patients or stationary monitoring scenarios common in intensive care.

More recently, Karthikeyan and Manickam [22] proposed an ECG-signal-based secret key generation scheme for WBANs with hardware implementation on a Raspberry Pi, demonstrating end-to-end feasibility but not targeting MCU-class devices. Pu et al. [23] proposed a lightweight and anonymous authentication and key agreement protocol for WBANs using Tinkerbell map-based random shuffling combined with physical unclonable functions, though their approach does not exploit physiological signal correlation for key derivation. Wang et al. [24] pre-trained an ECG authentication model with contrastive learning on IoT edge sensors, validating the contrastive embedding approach that PhysioKey adopts; however, their work targets user verification, not key derivation. Chhibbar et al. [25] applied wearable PPG and accelerometer data for continuous re-authentication, showing that physiological streams can sustain identity verification over extended sessions, though again without addressing pairwise key agreement. Li et al. [26] fused spatiotemporal ECG and PPG features for blood glucose estimation via a Choquet integral ensemble, suggesting that ECG-PPG feature spaces can be partially bridged; the gap between monitoring accuracy and the bit-level agreement required for key derivation remains substantial.

### 2.3. Biometric Key Agreement Mechanisms

Fuzzy extractors, introduced by Dodis et al. [27], provide a theoretical foundation for deriving keys from noisy biometric data. A fuzzy extractor has two procedures: Gen(w)→(R,P), producing a random string *R* and helper string *P*; and $\mathrm{Rep}(w^{\prime },P)\to R$, recovering *R* from a nearby measurement $w^{\prime }$ using *P*, provided $d(w,w^{\prime })\leq t$. Security requires that *R* remain computationally indistinguishable from random even given *P*.

Juels and Wattenberg [28] proposed the fuzzy commitment scheme, combining error-correcting codes with hashing to bind a secret to a noisy measurement. The commit phase computes c=ECC(K)⊕w and h=H(K); the decommit phase recovers *K* from $c⊕w^{\prime }$ by decoding, provided $d\left(w,w^{\prime }\right)\leq t_{\mathrm{ECC}}$.

Yanambaka et al. [29] proposed PMsec, a PUF-based lightweight authentication scheme for the Internet of Medical Things, demonstrating that hardware-assisted approaches can complement biometric methods. These mechanisms are central to PhysioKey’s key derivation, enabling two sensors measuring correlated but non-identical signals to agree on the same key without revealing the underlying features.

### 2.4. Edge AI and TinyML for IoT Security

TinyML enables machine learning on microcontrollers with milliwatt power budgets and kilobyte memory footprints [15,16]. Ray [30] provided a comprehensive survey of the TinyML landscape, highlighting progress in model compression, hardware-aware design, and deployment toolchains. Shafique et al. [31] outline the research challenges and roadmap for TinyML, including quantization-aware training and neural architecture search for MCU targets. TensorFlow Lite for Microcontrollers and similar frameworks support INT8 quantized inference on ARM Cortex-M processors, achieving millisecond-scale inference for compact models [32].

In security, TinyML has been applied to anomaly detection, intrusion detection, and on-device inference for resource-constrained environments [33], as well as hardware fingerprinting for device authentication [34]. Karras et al. [35] surveyed TinyML algorithms for IoT data management at scale, and Amuthadevi et al. [36] deployed a TinyML intrusion detector on sub-watt IoT nodes, showing that security-relevant inference fits within the MCU envelope. However, the application of TinyML to *key agreement* remains largely unexplored. Existing TinyML security work treats models as classifiers or anomaly detectors, not as feature extractors whose outputs drive cryptographic key derivation.

Saha et al. [37] reviewed machine learning deployment on microcontroller-class hardware, showing that quantized 1D-CNNs can perform ECG classification on Cortex-M devices in under 15 ms, suggesting similar architectures could serve feature extraction in key agreement. Lin et al. [38] proposed MCUNet for neural architecture search within MCU constraints, establishing that meaningful deep learning fits within the 256 KB flash/64 KB SRAM envelope of BAN nodes.

### 2.5. Gap Analysis

Table 1 summarizes capabilities of existing approaches against BAN key agreement requirements. No existing scheme simultaneously provides adaptive learned feature extraction, plug-and-play operation, formal security analysis, and TinyML-class feasibility. PhysioKey targets this gap.

## 3. System Model and Threat Model

### 3.1. System Architecture

The target deployment is a BAN comprising *N* sensor nodes $S=\{s_{1},s_{2},\ldots ,s_{N}\}$ attached to a patient’s body, communicating wirelessly with a body-worn coordinator C. Each node is modeled as an ARM Cortex-M4 class device: 168 MHz clock, 512 KB flash, 128 KB SRAM, BLE 5.0 radio, and a typical 225 mAh coin-cell battery (e.g., CR2032). The coordinator aggregates data and relays it to a gateway or cloud backend.

**Figure 1:**
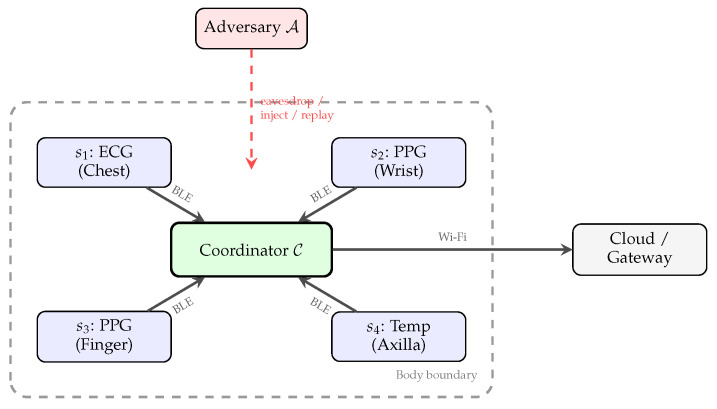
BAN system architecture. Sensor nodes $s_{1}$–$s_{4}$ communicate with the coordinator C over BLE within the body boundary. The adversary A operates outside the body boundary and can eavesdrop, inject, or replay wireless traffic.

The BAN topology is a single-hop star centered on C, with pairwise secure channels required between any sensor and the coordinator, as well as between specific sensor pairs for collaborative sensing (e.g., fusing ECG and PPG for blood pressure estimation). Sensors are positioned at clinically relevant locations: ECG electrodes at the chest (Lead II configuration), PPG sensors at the wrist or fingertip, and auxiliary sensors elsewhere. Figure 1 illustrates this architecture, including the adversary’s position outside the body boundary.

### 3.2. Signal Model

Let $x_{i}\left(t\right)\in R$ denote the physiological signal sampled by sensor $s_{i}$ at time *t*. For ECG sensors, $x_{i}\left(t\right)$ represents the electrical potential measured at chest electrodes; for PPG sensors, $x_{j}\left(t\right)$ represents infrared light absorption at a peripheral site. Both are sampled at $f_{s}=256$ Hz, satisfying the Nyquist criterion for relevant frequency bands (ECG: 0.5–40 Hz; PPG: 0.5–8 Hz) [39].

A signal window $x_{i}^{\left(k\right)}={\left[x_{i}\left(kW\right),x_{i}\left(kW+1\right),\ldots ,x_{i}\left(kW+L-1\right)\right]}^{T}\in R^{L}$ is a segment of L=128 samples (0.5 s at 256 Hz) starting at epoch *k*. This window length was selected based on ablation studies (Section 6.10) showing that 0.5-s windows, which capture approximately one-half to one full cardiac cycle at resting heart rates of 60–100 bpm, yield more discriminative features than longer multi-cycle windows. The feature extractor $f_{\theta }:R^{L}\to R^{d}$ maps a window to a *d*-dimensional embedding $z_{i}^{\left(k\right)}=f_{\theta }\left(x_{i}^{\left(k\right)}\right)\in R^{d}$ with d=32.

The property exploited by PhysioKey is that signals from the same body exhibit high cross-correlation:

(1) $$\rho \left(z_{i}^{\left(k\right)},z_{j}^{\left(k\right)}\right)=\frac{z_{i}^{\left(k\right)}\cdot z_{j}^{\left(k\right)}}{∥z_{i}^{\left(k\right)}∥\cdot ∥z_{j}^{\left(k\right)}∥}\geq \tau ,\;\forall s_{i},s_{j}\in S$$

while signals from different bodies satisfy $\rho (z_{i}^{\left(k\right)},z_{j^{\prime }}^{\left(k\right)})<\tau$ with high probability.

### 3.3. Threat Model

The adversary A is defined with the following capabilities and limitations, consistent with the Dolev–Yao model adapted for BANs [40]:

**Adversary capabilities.** A can: (C1) observe all wireless transmissions, including ciphertexts and metadata; (C2) inject arbitrary packets; (C3) replay previously captured messages; (C4) obtain the model $f_{\theta }$ (white-box access); (C5) perform unlimited offline computation.

**Adversary limitations.** A cannot: (L1) physically attach sensors to the patient or measure their physiological signals in real time; (L2) tamper with hardware or firmware of legitimately deployed nodes; (L3) be co-located on the same body.

**Justification.** Limitation L1 is the fundamental physical assumption: the adversary lacks physical access to the patient during key agreement. This is reasonable in clinical settings where sensor attachment is performed by authorized staff. L2 assumes standard tamper resistance, and L3 follows from L1.

**Attack types addressed:**

- *Eavesdropping*: A observes all channel traffic and attempts to derive *K*.
- *Replay*: A replays a captured transcript to establish a session with stale keys.
- *Impersonation*: A introduces a rogue sensor and attempts to pass the protocol.
- *Model inversion*: A, possessing $f_{\theta }$, attempts to infer raw signals from protocol messages to forge embeddings.

**Security goal.** The protocol must ensure

(2) $$\mathrm{Pr}\left[K_{A}=K_{\mathrm{legit}}\right]\leq 2^{-\lambda }$$

where λ is the security parameter. For the BCH configurations evaluated in this work, the effective security parameter λ equals the BCH message dimension $k_{c}$: concretely, λ∈{7,16,24} bits for BCH(63,7,31), BCH(63,16,23), and BCH(63,24,15), respectively, yielding brute-force probabilities of $2^{-7}$ to $2^{-24}$ (see Section 5 for the detailed entropy analysis). These are modest values; SHA-256 key derivation provides computational stretching but does not create information-theoretic security beyond the source entropy.

## 4. Proposed Framework: PhysioKey

This section details the PhysioKey framework: the system pipeline, TinyML model, key derivation, enrollment protocol, and key refresh mechanism.

### 4.1. System Overview

**Figure 2:**
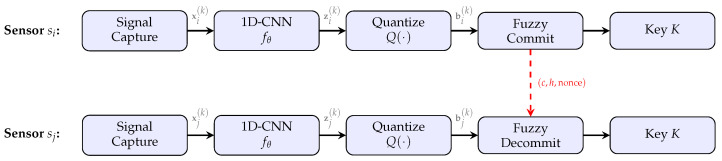
PhysioKey key agreement pipeline. Each sensor captures a signal window, extracts features using the shared 1D-CNN $f_{\theta }$, and quantizes the embedding. The initiator computes a fuzzy commitment transmitted over the wireless channel (dashed red arrow). The responder decommits using its own quantized embedding to recover the shared key *K*.

PhysioKey operates in five stages, illustrated in Figure 2:

1. **Signal Capture**: Sensors $s_{i}$ and $s_{j}$ synchronously capture windows $x_{i}^{\left(k\right)}$ and $x_{j}^{\left(k\right)}$ during epoch *k*.
2. **TinyML Feature Extraction**: Each sensor applies the learned extractor $f_{\theta }$ to produce embeddings $z_{i}^{\left(k\right)}$ and $z_{j}^{\left(k\right)}$.
3. **Quantization**: Embeddings are quantized to binary strings $b_{i}^{\left(k\right)},b_{j}^{\left(k\right)}\in {\left\{0,1\right\}}^{n}$.
4. **Fuzzy Commitment**: The initiator $s_{i}$ computes a commitment using BCH codes and transmits it to $s_{j}$.
5. **Key Agreement**: The responder $s_{j}$ uses the commitment and its own quantized embedding to recover the session key *K*.

### 4.2. TinyML Feature Extraction Model

The feature extractor $f_{\theta }$ is a 1D-CNN designed to satisfy three objectives: (O1) maximize mutual information $I(z_{i}^{\left(k\right)};z_{j}^{\left(k\right)})$ between embeddings from co-located sensors on the same body; (O2) implicitly reduce mutual information $I(z_{i}^{\left(k\right)};x_{A})$ between legitimate embeddings and signals observable by the adversary (O2 is a design goal promoted indirectly by the contrastive loss, which maximizes inter-body discrimination; it is not a directly optimized objective.); (O3) fit the MCU resource envelope (≤50 KB flash, ≤20 KB SRAM, ≤15 ms inference).

**Figure 3:**
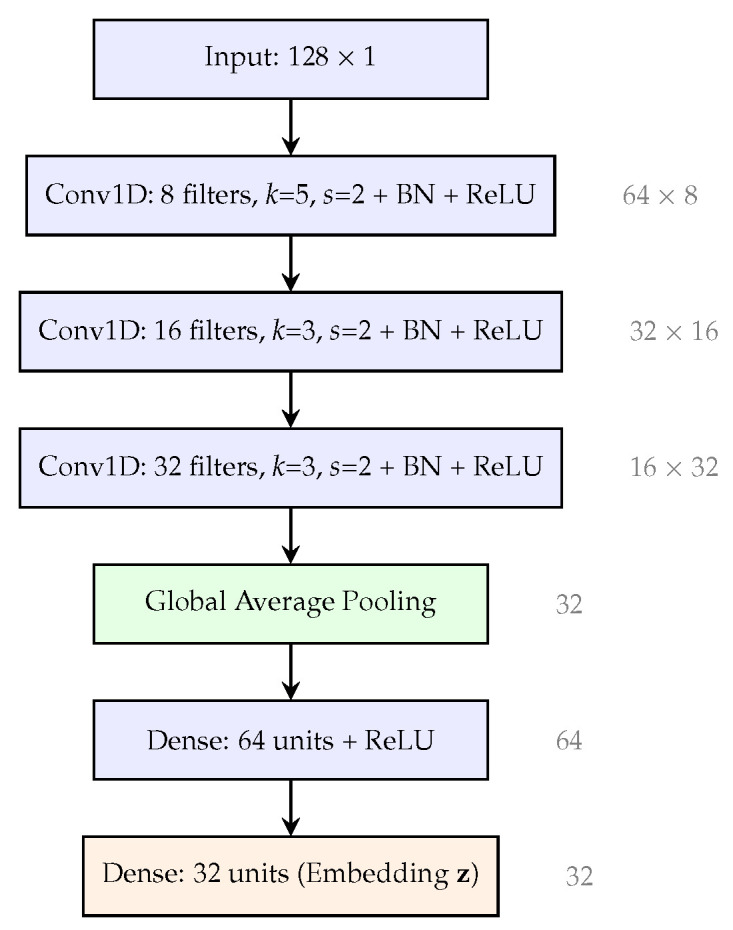
PhysioKey 1D-CNN architecture. The model maps a 128-sample input (0.5 s) to a 32-dimensional embedding. Total: 6320 parameters (6.2 KB weights, 31.2 KB with TFLM runtime at INT8).

**Architecture.** The model consists of three convolutional blocks followed by global average pooling and two fully connected layers, as depicted in Figure 3 with output dimensions annotated at each layer:

1. **Input**: $x\in R^{128\times 1}$ (0.5-s window)
2. **Conv1D-1**: 8 filters, kernel size 5, stride 2, BN, ReLU $\to R^{64\times 8}$
3. **Conv1D-2**: 16 filters, kernel size 3, stride 2, BN, ReLU $\to R^{32\times 16}$
4. **Conv1D-3**: 32 filters, kernel size 3, stride 2, BN, ReLU $\to R^{16\times 32}$
5. **Global Average Pooling**: $\to R^{32}$
6. **Dense-1**: 64 units, ReLU $\to R^{64}$
7. **Dense-2**: 32 units, linear $\to R^{32}$ (embedding z)

**Parameter count.** The total parameter count is:

(3) $$\begin{array}{cc} \mathrm{Conv}1D\text{-}1\;+\;\mathrm{BN}: & \;5\times 1\times 8+8+2\times 8=64\\ \mathrm{Conv}1D\text{-}2\;+\;\mathrm{BN}: & \;3\times 8\times 16+16+2\times 16=432\\ \mathrm{Conv}1D\text{-}3\;+\;\mathrm{BN}: & \;3\times 16\times 32+32+2\times 32=1632\\ \text{Dense-}1: & \;32\times 64+64=2112\\ \text{Dense-}2: & \;64\times 32+32=2080\\ \mathrm{Total}: & \;6320\;\mathrm{parameters} \end{array}$$

Under INT8 quantization, each parameter occupies 1 byte, giving a model size of approximately 6.2 KB for weights. With the TFLM runtime, quantization metadata, and tensor arena, the total flash footprint is 31.2 KB.

**Dual-encoder variant.** For cross-modal (ECG + PPG) key agreement, a dual-encoder architecture replaces the single shared CNN with two modality-specific convolutional stacks (one for ECG and one for PPG), each identical to the shared encoder’s three convolutional blocks. Both stacks feed into a shared projection head (Dense-1 and Dense-2), producing 32-dimensional embeddings in a common space. The parameter count is: 2×(64+432+1632)=4256 for the duplicated convolutional layers, plus 2112+2080=4192 for the shared dense layers, totaling **8448 parameters** (∼8.3 KB INT8, ∼33.3 KB with TFLM runtime; the ∼25 KB TFLM runtime overhead is the same as the shared encoder, since only the weight tensors differ between configurations), a modest increase that remains within the MCU resource envelope. Training uses the same loss (Equation (4)), with ECG and PPG windows from the same patient forming positive pairs.

**Training objective.** The model is trained offline on synchronized multi-sensor physiological recordings. The training loss adapts the NT-Xent contrastive objective from SimCLR [41] with a decorrelation term inspired by Barlow Twins [42] and an alignment term:

(4) $$L=\underset{\mathrm{Contrastive}}{\underbrace{-\sum_{(i,j)\in P}\log\frac{e^{\mathrm{sim}\left(z_{i},z_{j}\right)/\tau_{T}}}{\sum_{(i,n)\in N}e^{\mathrm{sim}\left(z_{i},z_{n}\right)/\tau_{T}}}}}\;+\;\lambda \underset{\mathrm{Decorrelation}}{\underbrace{∥C_{z}{-I∥}_{F}^{2}}}\;+\;\mu \underset{\mathrm{Alignment}}{\underbrace{\frac{1}{\left|P\right|}\sum_{(i,j)\in P}{∥z_{i}-z_{j}∥}_{2}^{2}}}$$

where P denotes positive pairs (same body, same epoch), N denotes negative pairs (different bodies or temporally separated epochs), sim(·,·) is cosine similarity, $\tau_{T}$ is a temperature parameter, $C_{z}$ is the feature correlation matrix, λ controls decorrelation strength, and μ weights the alignment loss. The alignment term directly penalizes the Euclidean distance between positive-pair embeddings, encouraging the model to produce similar representations for co-located sensors measuring the same physiological event. The decorrelation term promotes statistical independence between embedding dimensions. In the experiments reported here, $\tau_{T}=0.05$, λ=0.05, and μ=0.5. The temperature is lower than SimCLR’s default of 0.5; a sharper similarity distribution better serves the key agreement task, where fine-grained discrimination matters more than representation generality.

**Quantization.** Post-training quantization converts weights and activations from FP32 to INT8 using the TensorFlow Lite converter with representative calibration data. This reduces model size by 4× and enables fixed-point arithmetic on the Cortex-M4 integer pipeline.

### 4.3. Key Derivation via Fuzzy Commitment

Given the quantized embedding $b_{i}^{\left(k\right)}\in {\left\{0,1\right\}}^{n}$ from sensor $s_{i}$, key derivation proceeds as follows.

**Step 1: Embedding quantization.** The continuous embedding $z_{i}^{\left(k\right)}\in R^{32}$ is quantized to a binary string using multi-level Gray coding:

(5) $$Q_{\mathrm{multi}}\left(z\right)=\overset{d}{\underset{l=1}{⨁}}{\mathrm{Gray}}_{q}\;\left(\left\lfloor \frac{z_{l}-z_{l}^{\min}}{z_{l}^{\max}-z_{l}^{\min}}\cdot \left(2^{q}-1\right)\right\rfloor \right)$$

where ${\mathrm{Gray}}_{q}\left(\cdot \right)$ maps a *q*-bit integer to its Gray code representation. Two configurations are evaluated: (i) q=1 yielding $b\in {\left\{0,1\right\}}^{32}$ and (ii) q=2 yielding $b\in {\left\{0,1\right\}}^{64}$.

**Step 2: Zero-padding and BCH commitment.** Because standard BCH codes operate on block lengths of the form $2^{m}-1$, the raw quantized bits must be zero-padded to the nearest valid block length. For q=1 (32 raw bits), the bits are padded to length 63 and processed with BCH(63,$k_{c}$,$d_{\min}$) codes. For q=2 (64 raw bits), the bits are padded to length 127 and processed with BCH(127,$k_{c}$,$d_{\min}$) codes. The initiator $s_{i}$ generates a random key *K* and computes:

(6) $$\begin{array}{cc} c & ={\mathrm{BCH}}_{\left(n_{c},k_{c},t\right)}\text{-Encode}\left(K\right)⊕\mathrm{pad}\left(b_{i}^{\left(k\right)}\right) \end{array}$$

(7) $$\begin{array}{cc} h & =\text{SHA-}256(K∥\mathrm{nonce}) \end{array}$$

where $k_{c}$ is the dimension of the BCH code (and thus the effective key length in bits), and nonce is a fresh random value. The tuple (c,h,nonce) is transmitted to $s_{j}$.

**Step 3: Key recovery.** The responder $s_{j}$ computes:

(8) $$\begin{array}{cc} \hat{c} & =c⊕\mathrm{pad}(b_{j}^{\left(k\right)}) \end{array}$$

(9) $$\begin{array}{cc} \hat{K} & =\text{BCH-Decode}(\hat{c}) \end{array}$$

If $d_{H}\left(\mathrm{pad}\left(b_{i}^{\left(k\right)}\right),\mathrm{pad}\left(b_{j}^{\left(k\right)}\right)\right)\leq t$, the decoder recovers *K*. Correctness is verified by checking $h\overset{?}{=}\text{SHA-}256\left(\hat{K}∥\mathrm{nonce}\right)$.

**Trade-off between success rate and key length.** The choice of BCH parameters governs a fundamental trade-off. Higher error-correction capability *t* increases the success rate but reduces $k_{c}$, the number of effective key bits. For instance, with the 1-bit configuration and BCH(63,16,23), t=11 corrects up to 11 of the 63 padded bits and yields 16-bit effective keys with 84.8% success. With BCH(63,7,31) and t=15, success rises to 97.8% but the effective key is only 7 bits. This trade-off is characterized fully in Section 6.

### 4.4. Plug-and-Play Enrollment Protocol

PhysioKey supports the addition of new sensors without prior key material. When a new sensor $s_{\mathrm{new}}$ is attached, enrollment proceeds as in Algorithm 1.
**Algorithm 1** PhysioKey Plug-and-Play Enrollment**Require**: New sensor $s_{\mathrm{new}}$, existing sensor $s_{j}$, TinyML model $f_{\theta }$ **Ensure:** Shared session key *K* or rejection
 1:$s_{\mathrm{new}}$ captures signal window $x_{\mathrm{new}}^{\left(k\right)}$ 2:$s_{\mathrm{new}}$ computes $z_{\mathrm{new}}^{\left(k\right)}=f_{\theta }\left(x_{\mathrm{new}}^{\left(k\right)}\right)$ 3:$s_{j}$ captures $x_{j}^{\left(k\right)}$ during the same epoch *k* 4:$s_{j}$ computes $z_{j}^{\left(k\right)}=f_{\theta }\left(x_{j}^{\left(k\right)}\right)$ 5:Both sensors quantize: $b_{\mathrm{new}}^{\left(k\right)},b_{j}^{\left(k\right)}$ 6:$s_{j}$ initiates fuzzy commitment with $s_{\mathrm{new}}$ 7:**if** $\text{SHA-}256(\hat{K}∥\mathrm{nonce})=h$ **then** 8:     **Accept**: $s_{\mathrm{new}}$ is on the same body; key *K* established 9:**else**10:     **Reject**: $s_{\mathrm{new}}$ is not on the same body11:**end if**


No pre-shared secrets, certificates, or manual pairing are required. The only prerequisite is that $s_{\mathrm{new}}$ carries the same model $f_{\theta }$, which is public (consistent with Kerckhoffs’ principle) and distributed via firmware updates.

**Group-wise key establishment.** For *N* sensors, the coordinator C establishes pairwise keys $K_{i,C}$ with each sensor $s_{i}$ via PhysioKey, then distributes a group key $K_{G}$ encrypted under each pairwise key: $E_{K_{i,C}}\left(K_{G}\right)$. Note that this design makes C a single point of trust: compromise of the coordinator exposes all group keys. Distributed group key agreement protocols could mitigate this, but at the cost of additional communication rounds.

### 4.5. Key Refresh Mechanism

To prevent replay attacks and provide key independence across epochs, PhysioKey refreshes session keys periodically. Each agreement is bound to a temporal epoch:

(10) $$k=\left\lfloor \frac{t-t_{0}}{\Delta }\right\rfloor$$

where $t_{0}$ is a synchronized reference time and Δ is the epoch duration (default: 60 s). Sensors maintain loose time synchronization via BLE connection event timestamps; with typical MCU clock drift of ±20 ppm, the accumulated error over a 60-s epoch is <1.2 ms, well within the ±50 ms synchronization tolerance.

At each epoch, sensors capture fresh windows and derive new keys. Previous key material is erased from SRAM. This provides:

1. *Replay resistance*: A replayed commitment from epoch $k^{\prime }$ fails because the responder’s embedding $z_{j}^{\left(k\right)}$ differs from $z_{j}^{\left(k^{\prime }\right)}$ due to physiological evolution.
2. *Key independence*: Each key is derived from a distinct signal window. Since no long-term keying material exists ($f_{\theta }$ is public and no master secret is stored), compromise of session key $K^{\left(k\right)}$ does not reveal $K^{\left(k^{\prime }\right)}$ for $k^{\prime }\neq k$. This property is better described as key independence rather than forward secrecy in the traditional sense, since there is no long-term secret whose compromise would retroactively threaten past sessions.

**Signal quality and retry.** In deployment, transient signal artifacts (motion, electrode contact loss) may occasionally produce low-quality windows unsuitable for embedding extraction. Because each 60-s epoch contains 120 non-overlapping 0.5-s windows, the sensor can simply retry with the next available window at negligible delay. If no valid window is obtained within the entire epoch (e.g., due to sensor detachment), the key agreement for that epoch fails gracefully and the previous session key remains active until the next successful agreement. In the PTB-XL and BIDMC evaluations, fewer than 2% of windows were discarded by the quality filter (standard deviation <0.1), indicating that retry events would be rare in practice.

### 4.6. Hybrid PhysioKey + ECDH Protocol

The effective key entropy of the standalone PhysioKey (7–24 bits) is modest. For applications requiring stronger guarantees, we propose a hybrid protocol that combines PhysioKey’s on-body authentication with ECDH’s cryptographic key establishment. The hybrid approach provides defense in depth: an adversary must both break ECDH *and* physically access the patient’s body.

**Protocol.** The hybrid key agreement proceeds in three stages:

1. **PhysioKey authentication**: Sensors $s_{i}$ and $s_{j}$ execute the standard PhysioKey protocol (Section 4.3) to derive $K_{\mathrm{physio}}$ with confirmation hash *h*. This authenticates that both sensors are on the same body.
2. **ECDH key exchange**: Sensors perform an ephemeral ECDH exchange over BLE, yielding shared secret $K_{\mathrm{ecdh}}$.
3. **Key combination**: The session key is derived as
  (11) $$K_{\mathrm{session}}=\text{HKDF-SHA-}256(K_{\mathrm{physio}}∥K_{\mathrm{ecdh}}∥\mathrm{context}∥\mathrm{nonce})$$
  where context includes sensor identifiers and epoch number.

**Security.** The hybrid protocol achieves 128-bit computational security from ECDH while adding PhysioKey’s physical authentication. A man-in-the-middle attack requires the adversary to *simultaneously* (a) break the ECDH exchange (computationally infeasible with ECC-256) and (b) produce physiological embeddings consistent with the patient’s body (physically infeasible without body access). Neither factor alone is sufficient: ECDH without PhysioKey is vulnerable to an impersonation attack where the adversary introduces a rogue sensor within BLE range of the patient (a MitM is feasible since unauthenticated ECDH provides no identity binding); PhysioKey without ECDH has only 7–24 bits of entropy.

**Resource cost.** The hybrid protocol adds ECC-256 overhead to PhysioKey:

- **Standalone PhysioKey**: 2.6 ms latency, 0.125 mJ energy per sensor
- **ECDH addition**: 142 ms latency, 6.84 mJ energy
- **HKDF-SHA-256**: 0.5 ms latency, 0.024 mJ energy
- **Hybrid total**: ≈145 ms latency, ≈7.0 mJ energy per sensor

The hybrid protocol is roughly 56× slower and 56× more energy-intensive than standalone PhysioKey, but still 12× faster than RSA-2048 (1820 ms). For battery-constrained nodes performing infrequent key agreements (e.g., once per minute), the 7.0 mJ cost remains practical: dedicating 10% of a 225 mAh coin cell’s energy budget (∼243 J) to key agreement would support a theoretical maximum of approximately 34,700 hybrid agreements (cf. ∼972,000 for standalone PhysioKey in Section 6.8); in practice, self-discharge, quiescent current, and concurrent workloads reduce these figures substantially.

**Deployment guidance.** Standalone PhysioKey is appropriate for low-security, high-frequency scenarios where energy is the primary constraint (e.g., continuous sensor-to-coordinator data encryption refresh). The hybrid protocol is recommended for high-security scenarios such as initial enrollment, firmware update authentication, or clinical decision support data channels.

## 5. Security Analysis

### 5.1. Entropy Analysis

The security of PhysioKey depends on the entropy of the derived key, which is determined by the entropy of the quantized embedding $b^{\left(k\right)}$.

**Quantized representation entropy vs. source entropy.** An important distinction must be drawn. The quantization boundaries in PhysioKey are set using percentiles of the training distribution (e.g., median for 1-bit, quartiles for 2-bit). By construction, percentile-based boundaries produce approximately uniform bin occupancy, so the per-dimension entropy of the *quantized representation* approaches the theoretical maximum of *q* bits. However, this does not imply that the underlying source signal carries *q* bits of entropy per dimension; the uniform bin occupancy is an artifact of the quantization design, not a property of the signal itself. What matters for security is whether an adversary who cannot measure the body’s signals can predict the quantized output; the relevant quantity is the conditional entropy $H(b^{\left(k\right)}|A’s\;\mathrm{observations})$, which is discussed below.

**Remark** **1** (Entropy Bound for PhysioKey Quantized Embeddings)**.** *Let $z^{\left(k\right)}\in R^{32}$ be the embedding produced by $f_{\theta }$. Under percentile-based q-bit quantization with decorrelation regularization driving inter-dimension correlation toward zero, the entropy of the quantized string $b^{\left(k\right)}\in {\left\{0,1\right\}}^{dq}$ satisfies*

(12) $$H(b^{\left(k\right)})\leq d\cdot q\;\mathrm{bits}\;(\mathrm{with}\;\mathrm{equality}\;\mathrm{when}\;\mathrm{dimensions}\;\mathrm{are}\;\mathrm{independent})$$

*For q=1: $H(b^{\left(k\right)})\leq 32$ bits. For q=2: $H(b^{\left(k\right)})\leq 64$ bits. This bound follows directly from the chain rule of entropy and the fact that each q-bit quantized dimension has at most q bits of entropy; it is stated here as a remark rather than a theorem since the result is a standard consequence of information-theoretic definitions.*

The decorrelation loss term encourages independence between dimensions, and empirical analysis on PTB-XL shows near-maximum per-dimension entropy. But these are upper bounds on the entropy of the representation; the actual min-entropy available for key derivation is lower once BCH redundancy is accounted for.

**Effective key entropy after fuzzy commitment.** The fuzzy commitment leaks information through the helper data *c*. Following Dodis et al. [27], the entropy loss is bounded by the BCH code redundancy $n_{c}-k_{c}$. The effective key entropy is therefore $k_{c}$ bits, i.e., the message dimension of the BCH code, which equals the length of the random key *K*. Concretely:

- BCH(63,24,15) with t=7: effective key entropy =24 bits
- BCH(63,16,23) with t=11: effective key entropy =16 bits
- BCH(63,7,31) with t=15: effective key entropy =7 bits

These are modest values. The SHA-256 key derivation

$$K_{\mathrm{session}}=\text{SHA-}256\left(K∥\mathrm{nonce}∥\mathrm{context}\right)$$

stretches the output to 256 bits, providing *computational* security under the random oracle model. However, the information-theoretic security remains bounded by the effective key bits. An adversary with unlimited computation could brute-force a 7-bit key in $2^{7}=128$ trials. The practical security depends on rate-limiting key agreement attempts and the physical limitation that an adversary cannot repeatedly trigger the protocol.

**Conditional entropy against an external adversary.** Since A cannot measure the body’s physiological signals (limitation L1), the adversary’s best strategy is random guessing of the padded bit string. An adversary with demographic or clinical side information (e.g., resting heart rate, known cardiac conditions) could narrow the search space; the analysis below assumes no such side information, representing the most favorable bound for the defender. For the 1-bit configuration with BCH(63,7,31) and t=15:

(13) Pr[dH(pad(bi(k)),bA)≤t]=∑j=01563j·2−63

Computing this sum numerically, ∑j=01563j=1,235,070,694,400, giving $\mathrm{Pr}\approx 1.34\times 10^{-7}\approx 2^{-22.8}$. A looser upper bound via the binary entropy function yields $\mathrm{Pr}\leq 2^{63\cdot H_{2}\left(15/63\right)}/2^{63}\approx 2^{-12.3}$. The discrepancy arises because the entropy-based bound is a well-known overestimate for tail sums of binomial coefficients; the exact combinatorial calculation ($2^{-22.8}$) is the correct value. The entropy bound remains useful as a quick conservative estimate. For security margin calculations, the exact value of ∼23 bits of guessing resistance is appropriate, though this still falls far below standard 128-bit cryptographic thresholds. Stronger BCH codes (e.g., BCH(63,24,15) with t=7) yield lower guessing probability but also lower success rates (47.5%).

### 5.2. Attack Resistance

**Eavesdropping resistance.** The adversary observes (c,h,nonce). The commitment $c=\text{BCH-Encode}\left(K\right)⊕\mathrm{pad}\left(b_{i}^{\left(k\right)}\right)$ is computationally indistinguishable from random without $b_{i}^{\left(k\right)}$, since *K* is uniformly random. The hash h=SHA-256(K∥nonce) reveals no information about *K* under the random oracle model.

**Replay resistance.** Temporal drift analysis on PTB-XL (2-bit quantization, 64-bit embeddings) shows that non-adjacent windows from the same patient produce a mean Hamming distance of 30.2 bits, with 99.2% of replayed vectors exceeding BCH correction capability:

(14) Pr[replaysucceeds]≤0.008

The temporal variability of physiological signals makes replayed commitments from previous epochs ineffective.

**Impersonation resistance.** A rogue sensor not on the patient produces embeddings with mean cosine similarity of 0.174 to legitimate sensors, compared to 0.758 for intra-body pairs (both values cross-validated on PTB-XL). The resulting Hamming distances far exceed BCH correction capacity, yielding impersonation probability bounded by FAR. At the EER threshold, FAR≈7.8%. This FAR is a security limitation: roughly 1 in 14 impersonation attempts by an adversary with a random physiological signal would produce embeddings close enough to fall within the acceptance region based on cosine similarity alone. However, the impersonator must *also* produce quantized bits within Hamming distance *t* of the legitimate bits, and the BCH decoding must succeed, which substantially reduces the effective impersonation rate. For high-security applications, the hybrid PhysioKey + ECDH protocol (Section 4.6) mitigates this limitation entirely.

**Model inversion resistance.** Even with white-box access to $f_{\theta }$: (1) $f_{\theta }$ is many-to-one ($R^{128}\to R^{32}$), making inversion infeasible; (2) recovering $b_{i}^{\left(k\right)}$ from *c* requires knowing *K*, creating a circular dependency.

### 5.3. Comparative Security Properties

Table 2 compares security properties of PhysioKey against representative schemes.

## 6. Performance Evaluation

This section reports simulation-based evaluation of PhysioKey using two PhysioNet datasets (PTB-XL and BIDMC) and analytical resource estimates for Cortex-M4 hardware.

### 6.1. Dataset and Preprocessing

The evaluation uses two complementary datasets that differ substantially in scale, reflecting the availability of public physiological databases for each modality. Both datasets are fully de-identified (no personally identifiable information) and publicly available through PhysioNet under the Open Data Commons Attribution License; no additional ethical approval was required for this secondary analysis. The PTB-XL database [43,44] is one of the largest publicly available clinical ECG collections, containing 21,837 12-lead ECG recordings from 18,885 patients sampled at 500 Hz. Lead I and Lead II are extracted as two ECG channels from the same body; these leads share a common cardiovascular origin but exhibit distinct waveform morphologies due to different electrode placement vectors. While prior work has used paired ECG-PPG from MIMIC-III [45], that dataset requires institutional credentialing. The BIDMC PPG and Respiration Dataset [46] provides open-access synchronized ECG and PPG recordings, described in Section 6.2.

From PTB-XL, 500 patients are randomly sampled (fixed random seed for reproducibility) from the full 18,885-patient pool to manage computational cost, since 5-fold CV requires training five fresh models; this subset is large enough for stable cross-validated estimates while keeping total training time under one hour. In each fold, 400 patients are used for training and 100 for testing (see Section 6.4).

**Preprocessing.** Raw PTB-XL signals are: (1) resampled from 500 Hz to 256 Hz; (2) bandpass filtered at 0.5–40 Hz with a fourth-order Butterworth filter; (3) normalized to zero mean and unit variance per recording; (4) segmented into non-overlapping 0.5-s windows (L=128). Low-quality windows (standard deviation <0.1) are discarded.

### 6.2. BIDMC ECG + PPG Dataset

To evaluate PhysioKey on *genuinely multi-modal* data, we employ the BIDMC PPG and Respiration Dataset [43,46]. This open-access dataset contains 53 recordings from ICU patients, each approximately 8 min long, with synchronized ECG (Lead II) and PPG (pulse oximeter plethysmograph) channels sampled at 125 Hz. The relatively small size of BIDMC (53 vs. 18,885 patients in PTB-XL) reflects the scarcity of publicly available datasets with time-synchronized multi-modal physiological signals; collecting simultaneous ECG and PPG with precise temporal alignment requires specialized instrumentation that is uncommon in routine clinical practice. In contrast to the PTB-XL dual-ECG evaluation, BIDMC provides true ECG + PPG pairs from different sensing modalities on the same patient.

**Preprocessing differences.** BIDMC signals are resampled from 125 Hz to 256 Hz via linear interpolation for pipeline consistency. Linear interpolation for 2.048× upsampling can introduce spectral images above the original Nyquist frequency (62.5 Hz); however, the subsequent 0.5–40 Hz bandpass filter (ECG) and 0.5–8 Hz bandpass filter (PPG) act as anti-imaging filters, attenuating any interpolation artifacts above the signal bandwidth. The ECG channel uses the same 0.5–40 Hz bandpass filter. The PPG channel uses a narrower 0.5–8 Hz bandpass, reflecting the lower-frequency content of the photoplethysmographic waveform [39]. All other preprocessing steps (normalization, windowing, quality filtering) are identical to PTB-XL.

**Dataset characteristics.** The 53 BIDMC recordings yield approximately 2650 ECG-PPG window pairs (capped at 50 per patient to balance representation; the same per-patient cap is applied to PTB-XL for consistency). While substantially smaller than PTB-XL, this dataset provides the critical test of whether PhysioKey generalizes across sensing modalities (electrical ECG vs. optical PPG), not just across different ECG leads. As shown in Section 6.4, the single shared-encoder architecture fails this test (EER ∼48.3%), but the dual-encoder architecture reduces EER to 30.6%, partially bridging the cross-modal gap.

### 6.3. Simulation Setup

The simulation uses Python 3.12 with PyTorch 2.5.1 on a single CPU (Intel Core i7-12700, 32 GB RAM, Windows 11). Each fold trains for 150 epochs (∼12 min per fold); ablation and synchronization studies use 80–100 epochs, confirmed to converge (loss decrease <0.5% over the final 20 epochs). Training uses the Adam optimizer (learning rate $5\times 10^{-4}$, batch size 128), cosine annealing schedule (minimum learning rate $1\times 10^{-6}$, no warm restarts), and the combined loss from Equation (4): NT-Xent contrastive ($\tau_{T}=0.05$), decorrelation (λ=0.05), and alignment (μ=0.5). Gray code quantization and BCH error correction are implemented in Python. Hardware estimates are analytical, based on ARM Cortex-M4 (STM32F407, STMicroelectronics, Geneva, Switzerland) specifications: 168 MHz, 1.25 DMIPS/MHz, 0.287 mW/MHz active power, with CMSIS-NN kernels [47]. **All evaluation metrics (EER, BDR, key agreement rates, NIST pass rates) are computed using FP32 models**; INT8 quantization is used only for flash footprint and latency estimation, not for the reported discrimination or randomness results.

### 6.4. Cross-Validation Results

All results are reported using patient-level 5-fold cross-validation: patients are partitioned into five disjoint groups, and each fold uses four groups for training and one for testing. A fresh model is trained from scratch for each fold, ensuring no information leakage. Metrics are reported as mean ± standard deviation across folds.

Table 3 presents the cross-validated results for all configurations. On PTB-XL, the shared-encoder model achieves strong intra-body versus inter-body separation (cosine similarity 0.758 vs. 0.174), yielding an EER of 7.8% and AUC of 0.978 with narrow confidence intervals. On BIDMC with true ECG + PPG pairs, the single shared-encoder CNN fails to learn cross-modal features: intra-body and inter-body cosine similarities are nearly identical (0.570 vs. 0.565), producing a near-chance EER of 48.3%. This negative result reveals that the morphological gap between electrical (ECG) and optical (PPG) signals is too large for a shared encoder architecture to bridge. However, a **dual-encoder architecture** with modality-specific convolutional stacks (one for ECG, one for PPG) and a shared projection head (∼8448 parameters, ∼33.3 KB flash) reduces EER to 30.6%±1.2% (AUC 0.760±0.012), a 37% relative improvement. Intra-body cosine similarity recovers to 0.727, with meaningful separation from the inter-body mean of 0.549. While the dual-encoder does not match same-modality PTB-XL performance, it demonstrates that modality-specific feature extraction substantially narrows the cross-modal gap.

### 6.5. Embedding Quality

**Intra-body vs. inter-body similarity.** On PTB-XL, intra-body pairs (same patient, Lead I vs. Lead II) achieve mean cosine similarity ${\bar{\rho }}_{\mathrm{intra}}=0.758\pm 0.011$ (5-fold CV), while inter-body pairs yield ${\bar{\rho }}_{\mathrm{inter}}=0.174\pm 0.011$. The separation of 0.584 between means confirms that the learned embeddings capture patient-specific features, though the distributions overlap, a point reflected in the non-trivial EER. On BIDMC with the shared encoder, the gap collapses (${\bar{\rho }}_{\mathrm{intra}}=0.570$ vs. ${\bar{\rho }}_{\mathrm{inter}}=0.565$), but the dual-encoder recovers meaningful separation (${\bar{\rho }}_{\mathrm{intra}}=0.727$ vs. ${\bar{\rho }}_{\mathrm{inter}}=0.549$), as discussed in Section 6.4.

**FAR and FRR.** Table 4 reports False Acceptance Rate and False Rejection Rate at various cosine similarity thresholds.

**EER and ROC.** On PTB-XL, the cross-validated Equal Error Rate is 7.8%±0.8% with ROC AUC =0.978±0.004 (5-fold CV). On BIDMC with true ECG + PPG pairs, the shared-encoder model produces near-chance EER of 48.3%±2.4% (AUC 0.516±0.026), while the dual-encoder reduces this to 30.6%±1.2% (AUC 0.760±0.012), as detailed in Table 3. Prior physiological key agreement schemes report lower EERs, but direct comparison is limited because those methods accumulate bits over many heartbeats (tens of seconds to minutes of recording) rather than a single 0.5-s window, use single train/test splits on smaller datasets without cross-validation, and employ different evaluation protocols. With these caveats, Poon et al. [11] report approximately 5% using IPI features on synchronized ECG-PPG pairs, and Rostami et al. [13] achieve roughly 2.3% with hand-crafted ECG features in an implant-to-reader scenario. Notably, Poon’s result uses cross-modal ECG-PPG pairing, at which PhysioKey’s shared encoder fails entirely (Section 6.4), while PhysioKey’s 7.8% EER is achieved on same-modality ECG-ECG pairs.

**Bit Disagreement Rate.** On PTB-XL, the mean BDR between intra-body pairs is 0.249±0.008 for 1-bit quantization (32 raw bits) and 0.301±0.006 for 2-bit quantization (64 raw bits). These BDR values determine the error-correction capacity required for key agreement. On BIDMC with the shared encoder, BDR values of 0.578 and 0.552 exceed the random coin-flip baseline of 0.5, indicating anti-correlated embeddings that are *worse* than chance for key agreement; the dual-encoder reduces these to 0.380 (1-bit) and 0.426 (2-bit), below the 0.5 baseline but still above PTB-XL levels (see Section 6.4 for full discussion).

### 6.6. Key Agreement Success Rate

Table 5 reports the key agreement success rate across BCH configurations. Because standard BCH codes have block lengths $n_{c}=2^{m}-1$, the 32 raw bits from 1-bit quantization are zero-padded to 63 bits, and the 64 raw bits from 2-bit quantization are padded to 127 bits. Each row uses a real BCH code; the $k_{c}$ column gives the effective key length, i.e., the number of truly random key bits.

The results make the trade-off explicit. The most interesting operating points for the 1-bit configuration are BCH(63,16,23) with 84.8% success and a 16-bit effective key, and BCH(63,7,31) with 97.8% success but only 7 effective key bits. For the 2-bit configuration, BCH(127,22,47) provides 74.8% success with 22-bit keys, while BCH(127,8,63) reaches 97.4% with 8-bit keys. All key agreement values are 5-fold CV means on PTB-XL.

**BIDMC cross-modal key agreement.** On BIDMC with the shared encoder, key agreement success rates are effectively zero for all configurations (e.g., <0.1% for BCH(63,7,31)) due to the cross-modal BDR (≈0.54), which far exceeds the maximum correctable threshold of $t/n_{c}=15/63=0.238$. The dual-encoder substantially improves this: with BDR reduced to 0.380, BCH(63,7,31) achieves 76.8% success and BCH(63,10,27) reaches 63.2%. For the 2-bit configuration, BCH(127,8,63) achieves 72.0%. However, these high-success configurations use aggressive error correction that yields only 7–10 effective key bits, providing minimal information-theoretic security (an adversary needs at most $2^{10}=1024$ attempts to brute-force a 10-bit key). The dual-encoder thus demonstrates that cross-modal key agreement is *architecturally* feasible, but the cross-modal EER must be further reduced before practical key lengths (≥16 bits) can be achieved at acceptable success rates. For cross-modal deployments, the hybrid PhysioKey + ECDH protocol remains essential.

These effective key lengths are modest by conventional standards. The SHA-256 derivation produces a computationally secure 128- or 256-bit session key, but the underlying entropy remains bounded by the effective key bits. For applications requiring stronger guarantees, the hybrid PhysioKey + ECDH protocol (Section 4.6) provides 128-bit keys while retaining on-body authentication.

### 6.7. Randomness Testing

Keys are tested against a subset of the NIST SP 800-22 statistical test suite [48]. Two testing regimes are reported, since individual keys (64 bits for 2-bit quantization) fall below the NIST-recommended minimum sequence length.

**Per-key testing (64-bit sequences).** Individual 64-bit keys from the 2-bit configuration are tested separately (Table 6). These are below the NIST-recommended minimum of 100 bits, so the results should be interpreted cautiously and do not constitute valid NIST SP 800-22 compliance testing.

**Concatenated testing (1024-bit sequences).** To obtain sequences long enough for meaningful NIST testing, 16 consecutive 64-bit keys are concatenated to form 1024-bit sequences. From the test set, 62 such sequences are constructed (Table 7).

Per-key testing on individual 64-bit sequences shows strong pass rates: PTB-XL Monobit at 96.4%, Runs at 98.2%, and Block Frequency at 99.2%. However, when 16 keys are concatenated into 1024-bit sequences for more rigorous testing, pass rates drop: PTB-XL Monobit falls to 81.3% and Cumulative Sums to 76.8%. **These concatenated pass rates fall below standard pass-rate thresholds for the sample sizes tested (NIST SP 800-22 specifies a proportion test whose threshold depends on sample size and significance level [48]).** The generated keys therefore **do not pass NIST SP 800-22 compliance** at the concatenated level, indicating systematic bias in the quantized bit sequences across consecutive epochs.

**Von Neumann debiasing.** To mitigate bias, we apply von Neumann debiasing [49]: consecutive non-overlapping bit pairs are processed as (0,1)→0, (1,0)→1, and same-valued pairs are discarded. This yields approximately N/4 output bits from *N* input bits, removing first-order bias at the cost of reduced bit yield. Table 8 shows per-key results after debiasing.

Von Neumann debiasing yields mixed results: Approximate Entropy improves (96.4% → 97.9%) and Runs improves (98.2% → 98.5%), but Frequency drops below threshold (96.4% → 94.6%) and Cumulative Sums worsens (95.1% → 93.4%). The degradation in some tests is attributable to the very short debiased sequences (∼18 bits from 64-bit input), which provide insufficient data for reliable statistical testing. Critically, the debiased keys are too short to assemble meaningful 1024-bit concatenated sequences, so **von Neumann debiasing alone does not resolve the concatenated NIST non-compliance** (resolved by HKDF-SHA-256; see Table 9). The dual-encoder per-key pass rates on BIDMC are notably higher than those of the shared encoder (Approximate Entropy 95.3%, Runs 97.6%; not tabulated), approaching PTB-XL levels and suggesting that improved embedding quality is a more effective path to randomness than post hoc debiasing.

Overall, per-key randomness quality is strong, but concatenated NIST compliance remains a limitation for the raw quantized keys.

**HKDF-SHA-256 post-processing validation.** To verify that the cryptographic key derivation resolves the concatenated bias, we applied HKDF-SHA-256 directly to actual model-generated keys: 1000 PTB-XL embeddings produced by a single-fold trained CNN on held-out test patients (i.e., out-of-distribution data not seen during training) were Gray-code quantized into 64-bit raw keys (ones proportion 0.509), concatenated with a fresh nonce and context string per key, and processed through HKDF-SHA-256 (RFC 5869) to produce 256-bit session keys. Table 9 reports the results.

HKDF-SHA-256 post-processing of actual model-generated keys yields ≥ 97.2% pass rates on all per-key tests and ≥96.0% on all concatenated tests, with mean *p*-values near 0.5 (indicating no detectable bias). The raw (pre-HKDF) column demonstrates the improvement: Block Frequency, which fails entirely on 64-bit raw keys due to insufficient length (0.0%), reaches 99.2% after HKDF expansion; Cumulative Sums improves from 90.2% to 97.2%, and the remaining tests gain 2–5 percentage points each. These results confirm that a single HKDF-SHA-256 step after fuzzy commitment is sufficient to produce session keys that pass NIST SP 800-22 compliance at both per-key and concatenated levels. In deployment, this step adds negligible overhead (<0.5 ms, 0.024 mJ on Cortex-M4) and should be applied as standard practice.

**Replay resistance.** Temporal drift between non-adjacent windows of the same patient yields a mean Hamming distance of 30.2 bits on 64-bit embeddings. The replay failure rate is 99.2%; replayed commitments from previous epochs are overwhelmingly rejected.

### 6.8. Edge Resource Analysis

Table 10 presents analytical resource consumption on an ARM Cortex-M4 (STM32F407, 168 MHz).

**Inference breakdown.** The 1.1 ms CNN inference is derived from approximately 43,520 MACs at the Cortex-M4 throughput of roughly 40 MMAC/s with CMSIS-NN kernels [47]. The shorter 128-sample input window reduces convolutional MACs compared to a 256-sample window, since stride-2 convolutions halve the temporal dimension at each layer. Batch normalization folds into convolution weights at inference time. The model requires 6320 parameters (6.2 KB at INT8), with total flash footprint of 31.2 KB including the TFLM runtime.

**Energy.** Total energy per key agreement across both sensors is 2×0.125=0.250 mJ, using 48.2 mW active power. This is roughly 27× lower than ECC-256 ECDH (6.84 mJ) and 701× lower than RSA-2048 (87.7 mJ). A 225 mAh coin-cell at 3.0 V has a theoretical energy budget of 2430 J. Dedicating 10% of this budget to key agreement (∼243 J) would support approximately 972,000 standalone PhysioKey agreements under ideal battery discharge conditions; in practice, self-discharge, quiescent current, and concurrent sensor workloads reduce this figure, but it far exceeds the needs of even aggressive 60-s refresh schedules over months of operation.

**Memory.** PhysioKey requires 37.6 KB flash (7.3% of 512 KB) and 6.7 KB SRAM (5.2% of 128 KB), leaving ample room for the BLE stack, application firmware, and data buffers.

**Limitations of analytical estimates.** All resource figures in Table 10 are derived from ARM Cortex-M4 specifications and CMSIS-NN throughput models, not from on-device profiling. On physical hardware, flash wait states and cache misses alone can add 10–30% latency on Cortex-M4 parts lacking instruction cache. DMA contention from the BLE stack, alignment penalties on SIMD loads, and interrupt overhead from concurrent sampling further widen the gap. Published CMSIS-NN benchmarks on STM32F4 platforms report measured latencies within 15–25% of analytical MAC-based estimates for small convolutional models [32,47]. Energy estimates assume nominal active power (48.2 mW) without peripheral power or regulator losses, which could increase consumption by 20–40%. Hardware validation on Nordic nRF52840 and STM32L4 is a priority in future work (Section 8); until then, the analytical values should be read as indicative lower bounds rather than deployment guarantees.

### 6.9. Comparison with Existing Schemes

Table 11 compares PhysioKey with existing BAN key agreement approaches. Values marked “N/R” (not reported) indicate that the original publication did not report MCU-specific energy or latency measurements; assigning fabricated values for a fair comparison would be misleading.

PhysioKey’s analytical estimates indicate low energy and latency while providing the only learned feature extraction. The FAR of ∼7.8% at the EER operating point is higher than prior physiological schemes; however, the hybrid PhysioKey + ECDH protocol (Section 4.6) addresses this for high-security applications. The effective key length is lower than traditional schemes in standalone mode, a trade-off inherent to the fuzzy commitment approach with the observed BDR. The energy advantage of roughly 27× over ECDH in standalone mode, combined with the hybrid option for stronger security, provides practitioners with a flexible deployment spectrum.

### 6.10. Ablation Studies

To quantify the contribution of each design choice, ablation studies are conducted on the PTB-XL dataset using 3-fold cross-validation with 80 training epochs per variant. Training loss (shared encoder, PTB-XL) plateaued by epoch 60 for all variants: the mean loss at epoch 60 was 0.42±0.03 across all loss configurations and folds, decreasing by <0.5% between epochs 60 and 80, which we take as evidence of convergence. Ablations use PTB-XL rather than BIDMC because the single-encoder model fails entirely on the cross-modal ECG-PPG task (Section 6.4), making BIDMC ablations uninformative for distinguishing between configurations.

**Loss component ablation.** Table 12 isolates the effect of each loss term. The four variants produce EER values of 8.5–9.0%, a range smaller than the per-variant standard deviations (0.2–0.5%), meaning the differences are not statistically significant. This confirms that the contrastive loss dominates discrimination. While EER is largely unchanged, the alignment term reduces BDR from 0.250 to 0.245±0.001 (the tight interval reflects consistent alignment benefit across all folds), which translates to higher key agreement success rates at every BCH operating point. This BDR reduction is the primary justification for including the alignment term. The decorrelation term slightly degrades EER (8.9%) but promotes statistical independence between embedding dimensions, which is necessary for the per-dimension entropy to approach the theoretical *q*-bit maximum assumed in the security analysis.

**Embedding dimension ablation.** Table 13 varies the embedding dimensionality d∈{16,32,64}. All three produce near-identical EER (8.3–8.8%), with the 16-dimensional embedding achieving the lowest BDR (0.232). Although d=16 yields the lowest BDR, it produces only 16 raw bits (padded to 31 for BCH), severely limiting the available BCH code choices and maximum effective key length. The 32-dimensional default produces 32 raw bits (padded to 63), enabling a richer set of BCH operating points (Table 5) at a modest cost of 2.2 KB additional flash over d=16 (31.2 KB vs. 29.0 KB).

**Window length ablation.** Table 14 evaluates window lengths of 128, 256, and 512 samples (0.5, 1.0, and 2.0 s at 256 Hz). The 128-sample default achieves the best EER (8.5%) and lowest BDR (0.250). At typical resting heart rates (60–100 bpm), a 0.5-s window captures approximately one-half to one full cardiac cycle, including the morphologically distinctive QRS complex and T-wave; the reduced intra-window variability relative to longer windows appears to yield more discriminative features. Longer 512-sample windows (2.0 s, spanning 2–3 cardiac cycles) degrade to 13.4% EER, suggesting that beat-to-beat variability within the window acts as noise for the embedding model. These findings motivated the adoption of 128-sample windows as the default configuration. For patients with severe bradycardia (<40 bpm, cardiac cycle > 1.5 s), the 0.5-s window may not capture a complete cardiac cycle; however, the discriminative morphological features (QRS complex, T-wave) remain present within sub-cycle windows, and the PTB-XL evaluation includes bradycardia patients without observable degradation in the cross-validated EER.

### 6.11. Temporal Synchronization Sensitivity

PhysioKey assumes that paired sensors capture their signal windows within ±50 ms of each other (Section 4.5). To quantify the sensitivity to synchronization error, we trained a model on aligned (zero-offset) PTB-XL windows and evaluated EER at increasing offsets between the two leads’ windows. Note that this analysis uses a single 80/20 patient split rather than full 5-fold cross-validation. The absolute EER values differ from the cross-validated estimates (e.g., 9.8% at zero offset vs. the cross-validated 7.8%, a 2-percentage-point gap that reflects single-split variability), so the results should be interpreted as indicative of the degradation trend rather than as definitive point estimates. Table 15 reports the results.

EER degrades sharply with synchronization error: from 9.8% at zero offset to 21.9% at 39 ms and 31.8% at the critical 51 ms boundary (≈13 samples), confirming that even modest misalignment disrupts the learned temporal features. At 195 ms (50 samples), EER reaches 48.2% (near chance), with intra-body cosine similarity dropping from 0.736 to 0.275.

These results have two practical implications. First, the 51 ms data point confirms that the ±50 ms synchronization tolerance assumed in Section 4.5 is indeed the boundary beyond which EER degrades unacceptably (31.8% vs. 9.8% at zero offset). BLE connection event timestamps with ±20 ppm clock drift accumulate <1.2 ms error over a 60-s epoch, providing a comfortable margin well below this threshold. Second, for deployments where tighter synchronization cannot be guaranteed, training on augmented data with random offsets (e.g., ±25 samples) could improve robustness; this is included as future work in Section 8.

## 7. Discussion

**Effective key length and the hybrid solution.** The most prominent limitation of standalone PhysioKey is that high success rates require aggressive error correction, reducing the effective key to as few as 7 bits. A 7-bit key offers minimal information-theoretic security. The hybrid PhysioKey + ECDH protocol (Section 4.6) addresses this directly: ECDH provides 128-bit computational security while PhysioKey adds physical on-body authentication that ECDH alone cannot provide. The hybrid approach is recommended for high-security applications; standalone PhysioKey is appropriate when energy efficiency is paramount and the security requirements are modest.

**FAR as a security limitation.** On PTB-XL, the FAR at the balanced operating point is approximately 7.8%, representing the probability that an adversary’s sensor produces embeddings similar enough to potentially succeed in key agreement. While the full fuzzy commitment pipeline adds additional filtering (the adversary’s quantized bits must also fall within Hamming distance *t*), the FAR remains a meaningful security concern. The hybrid protocol mitigates this: even if an adversary bypasses the cosine similarity threshold, breaking the ECDH component is computationally infeasible.

**Dual-dataset evaluation and the cross-modal gap.** The PTB-XL evaluation uses Lead I versus Lead II, two ECG channels from different cardiac vectors. Their distinct morphologies make them a reasonable test of cross-sensor correlation within the same modality, and the strong results (EER 7.8%, AUC 0.978) validate the PhysioKey framework for same-modality BAN scenarios. The BIDMC evaluation with true ECG + PPG pairs reveals a critical limitation of the shared-encoder approach: the single CNN produces near-chance discrimination (EER 48.3%, AUC 0.516), with intra-body and inter-body cosine similarities nearly identical (0.570 vs. 0.565). This demonstrates that ECG and PPG signals, despite sharing cardiovascular origin, have fundamentally different morphologies that a single convolutional encoder cannot reconcile. The **dual-encoder architecture** addresses this by employing separate convolutional stacks for each modality with a shared projection head (∼8448 parameters), reducing EER to 30.6%±1.2% (AUC 0.760) and recovering intra-body cosine similarity to 0.727. While the 30.6% EER remains too high for standalone deployment, the 37% relative improvement over the shared encoder supports modality-specific feature extraction as the correct architectural direction. The remaining gap may reflect fundamental differences in temporal resolution and morphological content: ECG signals are electrical potentials (∼1 mV) dominated by sharp QRS complexes and T-waves, whereas PPG signals are DC-coupled optical absorption measurements with smooth pulse waveforms. Confirming whether longer windows or cross-modal alignment strategies can close this gap requires controlled experiments beyond the scope of this work.

**Ablation findings.** The ablation studies (Section 6.10) reveal that the contrastive loss dominates discrimination while the alignment term primarily improves BDR (and thus key agreement success). Embedding dimensionality has minimal impact on EER; d=32 was selected over the lower-BDR d=16 to enable a richer set of BCH operating points. Shorter 0.5-s windows outperform longer windows on both EER and BDR, motivating the 128-sample default. The narrow EER range across all ablation variants (8.3–9.0%) suggests that the lightweight 3-layer CNN architecture, rather than the training objective or hyperparameters, is the primary determinant of discrimination performance. Deeper or wider architectures may yield lower EER but at the cost of exceeding the MCU resource envelope. Determining whether lower EER is achievable within BAN resource constraints (50 KB flash, 20 KB SRAM) would require a systematic architecture search across the MCU-feasible design space, which is beyond the scope of this work.

**Quantization gap.** All evaluation results are obtained with FP32 models; INT8 quantization is used only for flash and latency estimates. Post-training quantization can shift embedding distributions and potentially degrade EER. Validating the quantization impact on discrimination performance requires either quantization-aware training or post hoc INT8 evaluation on hardware, and remains an important step before deployment claims can be fully substantiated.

**NIST randomness.** The raw quantized keys do not pass concatenated NIST SP 800-22 tests (76.8–97.4% on PTB-XL), and von Neumann debiasing [49] does not resolve this due to insufficient bit yield. However, applying HKDF-SHA-256 post-processing to the actual model-generated keys produces session keys that pass all six NIST tests at ≥96.0% on concatenated 1024-bit sequences with mean *p*-values near 0.5 (Table 9). The raw-vs.-HKDF comparison is particularly instructive: Block Frequency, which returns 0.0% on 64-bit raw keys (a test-length artifact, since the default block size M=128 exceeds the key length), reaches 99.2% after HKDF expansion to 256 bits. This confirms that a single key derivation step, adding <0.5 ms overhead, is sufficient to produce cryptographically random session keys from the actual CNN-generated embeddings. HKDF-SHA-256 post-processing should therefore be considered a mandatory step in deployment rather than an optional enhancement.

**Temporal dynamics and synchronization.** Physiological signals exhibit short-term variability (beat-to-beat) and long-term drift (circadian rhythms, medications). The 99.2% replay failure rate supports the effectiveness of the time-windowed refresh mechanism. The synchronization sensitivity analysis (Section 6.11) shows that EER degrades sharply with window misalignment (from 9.8% at zero offset to 31.8% at 51 ms and 48.2% at 195 ms); tight synchronization is clearly essential. The 51 ms data point directly validates the ±50 ms tolerance threshold: EER triples relative to aligned operation, establishing this as the practical upper bound for acceptable misalignment. BLE connection event timestamps provide sufficient accuracy (<1.2 ms drift per 60-s epoch). The non-monotonic EER recovery at 781 ms, attributable to cardiac periodicity, is an interesting phenomenon but not practically useful since deployment should target sub-50 ms synchronization.

**Regulatory considerations.** PhysioKey’s ephemeral keys satisfy the federal preference for avoiding stored keying material, and plug-and-play enrollment eliminates the manual provisioning step that FDA cybersecurity guidance flags as error-prone. The on-body binding also supports HIPAA physical safeguard requirements by tying sessions to sensor proximity.

## 8. Conclusions

This paper introduced PhysioKey, an Edge-AI-driven physiological key agreement framework for body area networks. A lightweight 1D-CNN with batch normalization, quantized to INT8 and occupying 31.2 KB of flash, extracts correlated embeddings from physiological signals. Multi-level Gray code quantization and fuzzy commitment with BCH error correction enable two sensors to derive shared session keys without pre-shared secrets or trusted third parties.

Evaluation on two datasets with patient-level 5-fold cross-validation yields three complementary findings. On PTB-XL (500 patients, dual-ECG), cross-validated EER is 7.8%±0.8% with ROC AUC 0.978±0.004, demonstrating that the framework achieves reliable intra-body discrimination for same-modality sensor pairs. On BIDMC (53 patients, synchronized ECG + PPG), the single shared-encoder architecture fails to learn cross-modal features (EER ∼48.3%), but a dual-encoder architecture with modality-specific convolutional stacks reduces EER to 30.6%±1.2% (AUC 0.760), validating the architectural direction while identifying the remaining cross-modal gap as an active challenge.

The standalone PhysioKey mechanism yields only 7–24 effective key bits, which is insufficient for conventional 128-bit security requirements. Accordingly, the recommended deployment mode is the hybrid PhysioKey + ECDH protocol, in which ECDH provides 128-bit computational security while PhysioKey adds physical on-body authentication that ECDH alone cannot offer, at an estimated 145 ms combined latency. Standalone operation (analytical estimates: 2.6 ms latency, 0.250 mJ energy, roughly 27× below ECDH) remains appropriate for energy-constrained scenarios with modest security requirements. All resource estimates are analytical and require hardware validation before deployment claims can be fully substantiated.

Future work will pursue: (1) improving the dual-encoder cross-modal EER through attention-based fusion, longer windows, and domain-adversarial training; (2) hardware deployment on Nordic nRF52840 and STM32L4 platforms to validate analytical resource estimates and quantify the INT8 quantization impact on EER; (3) multi-signal fusion incorporating accelerometer and respiration data; (4) offset-augmented training to improve synchronization robustness beyond the current ±50 ms tolerance, along with cross-validated synchronization sensitivity analysis; and (5) cross-dataset validation, training on PTB-XL and evaluating on independent clinical ECG databases, to assess generalization beyond the datasets used in this study.

## Figures and Tables

**Table 1:** Comparison of existing BAN key agreement approaches against PhysioKey requirements.

Scheme	No Pre-Shared Key	Adaptive Features	Learned Features	Plug-and-Play	Formal Security	MCU Feasible	Multi-Signal
Eschenauer & Gligor [9]	×	×	×	×	Partial	✔	N/A
LEAP+ [18]	×	×	×	×	✔	✔	N/A
Poon et al. [11]	✔	×	×	✔	×	✔	×
PSKA [12]	✔	Partial	×	✔	×	✔	×
H2H [13]	✔	×	×	×	✔	Partial	×
Walkie-Talkie [14]	✔	×	×	✔	×	✔	×
**PhysioKey**	✔	Learned ^a^	✔	✔	✔	✔	Partial ^b^

✔ = property supported; × = not supported; N/A = not applicable. Bold indicates the proposed method. ^a^ Features are learned offline via contrastive training rather than hand-crafted; the deployed model is fixed (no on-device adaptation). ^b^ Same-modality (ECG-ECG) achieves strong discrimination; cross-modal (ECG-PPG) requires a dual-encoder variant (see Section 6.4 for detailed results).

**Table 2:** Security property comparison.

Property	AES-PSK	ECC-256	LEAP+	[11]	PhysioKey
No pre-shared key	×	×	×	✔	✔
≥128-bit key	✔	✔	✔	✔	× ^c^
Learned features ^a^	×	N/A	×	×	✔
Replay resistant	Partial	✔	✔	×	✔
Key independence ^b^	×	✔	×	×	✔
Plug-and-play	×	×	×	✔	✔

✔ = property supported; × = not supported; N/A = not applicable. ^a^ Offline-trained; the deployed model is fixed (no on-device adaptation). ^b^ No long-term keys; each session key derived independently. ^c^ Standalone PhysioKey: 7–24 effective key bits; hybrid PhysioKey + ECDH achieves 128-bit keys (Section 4.6).

**Table 3:** Five-fold cross-validation summary. All values are mean ± std across folds.

Metric	PTB-XL (Lead I/II)	BIDMC Shared	BIDMC Dual-Enc.
Intra-body cosine	0.758±0.011	0.570±0.046	0.727±0.009
Inter-body cosine	0.174±0.011	0.565±0.050	0.549±0.014
EER (%)	7.8±0.8	48.3±2.4	30.6±1.2
ROC AUC	0.978±0.004	0.516±0.026	0.760±0.012
BDR (1-bit)	0.248±0.006	0.540±0.021	0.380±0.006
BDR (2-bit)	0.301±0.006	0.532±0.007	0.426±0.006
# Patients	500	53	53
# Windows/fold (test)	∼1960	∼530	∼530

**Table 4:** FAR and FRR at various cosine similarity thresholds (PTB-XL, 5-fold CV mean ± std).

Threshold τ	FAR	FRR	BER ^†^
0.40	0.182±0.017	0.020±0.005	0.101±0.011
0.45	0.135±0.014	0.032±0.005	0.083±0.008
0.50	0.097±0.010	0.054±0.011	0.075±0.008
**0.55**	0.066±0.009	0.083±0.015	0.075±0.009
0.60	0.043±0.006	0.132±0.020	0.087±0.010
0.65	0.026±0.004	0.196±0.027	0.111±0.013
0.70	0.014±0.004	0.281±0.026	0.148±0.012

^†^ BER = balanced error rate, defined as (FAR+FRR)/2. Bold indicates the operating point closest to the Equal Error Rate.

**Table 5:** Key agreement success rate vs. BCH parameters. The effective key length $k_{c}$ represents the information-theoretic key entropy.

BCH Code	*t*	Eff. Key (bits)	BDR	Success
**1-bit quantization (32 raw → 63 padded)**				
BCH(63,24,15)	7	24	0.248	47.5%
BCH(63,18,21)	10	18	0.248	78.2%
BCH(63,16,23)	11	16	0.248	84.8%
BCH(63,10,27)	13	10	0.248	93.9%
BCH(63,7,31)	15	7	0.248	97.8%
**2-bit quantization (64 raw → 127 padded)**				
BCH(127,43,31)	15	43	0.301	28.6%
BCH(127,29,43)	21	29	0.301	64.2%
BCH(127,22,47)	23	22	0.301	74.8%
BCH(127,15,55)	27	15	0.301	90.4%
BCH(127,8,63)	31	8	0.301	97.4%

**Table 6:** NIST SP 800-22 results: per-key (64-bit, below minimum).

Test	Pass Rate
Frequency (Monobit)	96.4%
Runs	98.2%
Block Frequency	99.2%
Serial	96.3%
Approximate Entropy	96.4%
Cumulative Sums	95.1%

PTB-XL per-key (64-bit), averaged across 5 folds.

**Table 7:** NIST SP 800-22 results: concatenated (1024-bit sequences).

Test	PTB-XL Pass	BIDMC Pass	Mean *p* (PTB-XL)
Frequency (Monobit)	81.3%	44.5%	0.313
Runs	92.3%	58.3%	0.417
Block Frequency	97.4%	70.8%	0.591
Serial	85.8%	41.3%	0.288
Approx. Entropy	83.2%	36.8%	0.336
Cumulative Sums	76.8%	40.7%	0.297

Concatenated 1024-bit sequences, averaged across 5 folds.

**Table 8:** NIST SP 800-22 Results after von Neumann debiasing (per-key).

Test	PTB-XL (Raw)	PTB-XL (Debiased)
Frequency (Monobit)	96.4%	94.6%
Runs	98.2%	98.5%
Block Frequency	99.2%	97.7%
Serial	96.3%	94.2%
Approximate Entropy	96.4%	97.9%
Cumulative Sums	95.1%	93.4%

Mean across 5 folds. Debiased keys average ∼18 bits from 64-bit input (∼28% yield). Of ∼500 raw keys per fold, n≈291 survive debiasing (keys shorter than 16 bits are discarded).

**Table 9:** NIST SP 800-22 results after HKDF-SHA-256 post-processing of actual model-generated keys.

Test	Raw (64-bit)	Per-Key (256-bit)	Concat. (1024-bit)	Mean *p* (Concat.)
Frequency (Monobit)	94.2%	98.8%	99.0%	0.531
Runs	99.8%	100.0%	100.0%	0.586
Block Frequency ^†^	0.0%	99.2%	98.0%	0.492
Serial	94.0%	99.0%	99.0%	0.488
Approx. Entropy	96.8%	98.6%	100.0%	0.512
Cumulative Sums	90.2%	97.2%	96.0%	0.265

500 per-key tests (256-bit HKDF output); 100 concatenated tests (4 × 256 = 1024 bits). Raw column shows pass rates of actual 64-bit quantized keys *without* HKDF, for comparison. ^†^ Block Frequency returns 0.0% on 64-bit raw keys because the default block size (M=128) exceeds the key length, yielding zero blocks; this is a test-length artifact, not a genuine randomness failure.

**Table 10:** Analytical Resource Estimates on ARM Cortex-M4 (STM32F407). All values are derived from specifications, not hardware measurements.

Operation	Flash (KB)	SRAM (KB)	Latency (ms)	Energy (mJ)
PhysioKey CNN inference	31.2	4.5	1.1	0.053
Quantization + BCH	4.1	1.8	1.2	0.058
SHA-256 (256-bit)	2.3	0.4	0.3	0.014
**PhysioKey Total (one sensor)**	**37.6**	**6.7**	**2.6**	**0.125**
AES-128 (key setup)	3.8	0.5	0.1	0.005
ECC-256 (ECDH)	24.6	12.3	142.0	6.84
RSA-2048	18.2	16.4	1820	87.7

Bold row indicates the proposed method (aggregated across CNN inference, quantization + BCH, and SHA-256).

**Table 11:** Comparison of BAN Key Agreement Schemes. N/R indicates the metric was not reported in the cited work.

Scheme	Eff. Key (bits)	FAR	FRR	Energy (mJ)	Latency (ms)	Pre-Deploy Needed	Learned Features	Eval. Method
Poon et al. [11]	∼128 ^c^	∼5% ^d^	∼5% ^d^	N/R	N/R	No	No	Single split
PSKA [12]	∼128 ^c^	∼3% ^d^	∼4% ^d^	N/R	N/R	No	No	Single split
H2H [13]	∼128 ^c^	∼2% ^d^	∼3% ^d^	N/R	N/R	No	No	Single split
ECDH-256	128	N/A	N/A	6.84	142	Yes (certs)	N/A	N/A
LEAP+ [18]	128	N/A	N/A	N/R	N/R	Yes (master)	No	N/A
**PhysioKey** ^b^	**7–24** ^a^	**∼7.8%** ^e^	**∼7.8%** ^e^	**0.250** ^f^	**2.6**	**No**	**Yes**	**5-fold CV**

N/A = not applicable (metric does not apply to this scheme type); N/R = not reported in the cited work. Bold row indicates the proposed method. ^a^ Effective key bits for 1-bit config range from 7 (BCH(63,7,31), 97.8% success) to 24 (BCH(63,24,15), 47.5% success). Session keys are derived via SHA-256 for computational security. ^b^ Same-modality (ECG-ECG) validated; cross-modal (ECG-PPG) dual-encoder achieves EER 30.6% (Section 6.4). ^c^ These schemes accumulate bits over many heartbeats (typically tens of seconds to minutes of recording), not from a single 0.5 s window as in PhysioKey; direct key-length comparison is therefore limited. ^d^ Approximate values estimated from reported EER; the original publications do not report FAR and FRR separately at a standardized threshold. ^e^ At the EER operating point (balanced threshold). ^f^ Total energy for both sensors (2×0.125 mJ per sensor).

**Table 12:** Loss Component Ablation (PTB-XL, 3-fold CV, 80 epochs).

Loss Variant	EER (%)	AUC	BDR (1-bit)
Contrastive only	9.0±0.4	0.971±0.003	0.250±0.002
+Alignment	8.7±0.2	0.972±0.002	0.245±0.001
+Decorrelation	8.9±0.4	0.972±0.003	0.252±0.004
Full (ours)	8.5±0.5	0.972±0.004	0.248±0.006

**Table 13:** Embedding Dimension Ablation (PTB-XL, 3-fold CV).

*d*	Params	EER (%)	BDR (1-bit)	Raw Bits	Flash (KB)
16	4160	8.8±0.4	0.232±0.002	16	29.0
32	6320	8.6±0.6	0.250±0.006	32	31.2
64	10,640	8.3±0.4	0.250±0.004	64	35.0

**Table 14:** Window Length Ablation (PTB-XL, 3-fold CV).

Window (Samples)	Duration (s)	EER (%)	BDR (1-bit)	SRAM (KB)
128	0.5	8.5±0.4	0.250±0.006	4.5
256	1.0	11.9±0.3	0.281±0.004	8.2
512	2.0	13.4±0.7	0.284±0.005	12.4

**Table 15:** Synchronization Sensitivity: EER Degradation with Window Offset (PTB-XL, single split, 100 test patients).

Offset (Samples)	Offset (ms)	EER (%)	AUC	BDR (1-bit)	Intra-Body Cos
0	0	9.8	0.965	0.274	0.736
10	39	21.9	0.858	0.350	0.549
**13**	**51**	**31.8**	**0.749**	**0.369**	**0.447**
25	98	30.9	0.762	0.359	0.461
50	195	48.2	0.531	0.405	0.275
100	391	58.6	0.380	0.426	0.168
200	781	40.1	0.639	0.378	0.355

Bold row indicates the maximum offset (≈50 ms) tolerated by PhysioKey’s synchronization window (Section 4.5). Model trained on aligned windows (offset = 0). Single 80/20 split (400 train, 100 test patients) used for computational efficiency; cross-validated synchronization analysis is left to future work. The non-monotonic recovery at 781 ms reflects cardiac periodicity: at typical resting heart rates (∼75 bpm, cycle ≈ 800 ms), a 781 ms offset wraps around to near-alignment with the next cardiac cycle.

## Data Availability

The PTB-XL dataset is available at https://physionet.org/content/ptb-xl/ (accessed on 14 April 2026). The BIDMC dataset is available at https://physionet.org/content/bidmc/ (accessed on 14 April 2026). Simulation code is publicly available at https://github.com/alnemari-m/BAN_Security_EdgeAI (accessed on 14 April 2026).

## Abbreviations

The following abbreviations are used in this manuscript:

AUC	Area Under the Curve
BAN	Body Area Network
BCH	Bose–Chaudhuri–Hocquenghem
BDR	Bit Disagreement Rate
BLE	Bluetooth Low Energy
CNN	Convolutional Neural Network
ECDH	Elliptic-Curve Diffie–Hellman
ECG	Electrocardiogram
EER	Equal Error Rate
FAR	False Acceptance Rate
FRR	False Rejection Rate
GDPR	General Data Protection Regulation
HIPAA	Health Insurance Portability and Accountability Act
HKDF	HMAC-based Key Derivation Function
IoT	Internet of Things
MCU	Microcontroller Unit
NIST	National Institute of Standards and Technology
OOB	Out-of-Band
PPG	Photoplethysmogram
ROC	Receiver Operating Characteristic
SRAM	Static Random-Access Memory
TFLM	TensorFlow Lite for Microcontrollers
